# Linking CD38 Cell Surface Expression to the Unique Calcium Flux Properties in Human Peripheral B Lymphocytes Following anti‐IgM and anti‐IgG Stimulation

**DOI:** 10.1155/jimr/1377217

**Published:** 2026-08-27

**Authors:** Viktória Temesfői, Péter Kaltenecker, Anna Nörenberg, Tímea Serény-Litvai, Ambrus Kaposi, Emese Mezősi

**Affiliations:** ^1^ Hungarian National Laboratory on Reproduction, University of Pecs, Pecs, Hungary, pte.hu; ^2^ Janos Szentagothai Research Centre, University of Pecs, Pecs, Hungary, pte.hu; ^3^ Department of Obstetrics and Gynaecology, University of Pecs Medical School, Clinical Centre, Pecs, Hungary, pte.hu; ^4^ Department of Programming Languages and Compilers, Faculty of Informatics, Eotvos Lorand University, Budapest, Hungary, elte.hu; ^5^ First Department of Internal Medicine, University of Pecs Medical School, Clinical Centre, Pecs, Hungary, pte.hu

**Keywords:** B cell activation, calcium flux, CD38, kinetic flow cytometry

## Abstract

CD38 serves as a marker for defining subsets of immune cells; however, being an ectoenzyme, it also plays a crucial part in modulating immunometabolic homeostasis, has immense potential to influence the microenvironment of tissues, functions as a prognostic indicator, as well as a therapeutic target. Despite the extensive information on CD38 and the scale of efforts in developing and optimizing therapies targeting various B cell‐related diseases, there is still ongoing fundamental research addressing its basic functions on healthy human B cells, which is well needed. Using an adapted methodology to measure and analyze intracellular Ca^2+^ concentration changes in B cells upon anti‐Ig activation, this study describes the possible effect of CD38 cell surface expression on the rapid Ca^2+^ flux response of different B cell subsets from healthy individuals, aiming to better understand the role and influence of CD38 on B cell behavior. Circulating B cell subpopulations bearing IgM type BCRs showed marked differences in their Ca^2+^ flux characteristics according to their respective expression levels of CD38, with the CD38^high^ transitional cells being visibly different from their transitional and naïve CD38^+^ and CD38^−^ counterparts, and the unswitched CD38^−^ cells being clearly distinct based on their Ca^2+^ flux pattern from the CD38^+^ cells in the same compartment, in a setting where CD38 expression was the sole differentiating factor among the subpopulations. The CD38^−^ unswitched cells had the highest maximum Ca^2+^ concentration among the IgM‐expressing populations achieved during anti‐IgM stimulation. IgG^+^ class‐switched memory cells were clustered uniformly by their activation patterns, showed strong responses to anti‐IgG stimulation, with no significant correlations to CD38 expression levels. While our research could not fully separate the functional effects of CD38, this work revealed descriptive data concerning the differential kinetic Ca^2+^ responses of circulating B cells, categorized by their CD38 expression levels.

## 1. Introduction

From cellular activation to cell‐fate decisions and long‐lived serological memory, intracellular calcium ion (Ca^2+^) fluxes are conveying information of the surface stimulus to create diverse outputs that manifest as physiological responses of the cells, specific to the initiating signal by translating them into amplitude, oscillation and perseverance of Ca^2+^ concentration [1, 2]. Subsequently, a series of proteins that interact with Ca^2+^ facilitate transcriptional regulation, ultimately governing the whole immune system in both health and disease [3]. Cluster of differentiation (CD) 38 contributes to this regulatory matrix by aiding Ca^2+^ release from the endoplasmic and endolysosomal stores via the byproducts of its NADase activity [4–6] and by engaging in supramolecular signaling complexes as a co‐receptor [7–9].

CD38 is a multifunctional single‐chain membrane glycoprotein, ~45 kDa in size, possessing receptor function, and a catalytic C‐terminal domain, identified as the main cellular nicotinamide adenine dinucleotide (NAD^+^) hydrolase in mammalian cells, with one transmembrane sequence, and a short, 20 amino acid N‐terminal domain [10, 11]. It is most highly expressed in tissues that have a role in hematopoiesis, such as bone marrow and lymph nodes, without being lineage restricted. The highest expression in human immune cells is detected in transitional B cells, antibody‐secreting cells (ASCs), natural killer (NK), and dendritic cells (DC) [12]. More than 80% of medullary thymocytes are CD38^+^, while the majority of circulating T cells are CD38^−^, and T cells in tissues are mainly positive for the marker. The molecule is found on the surface of circulating monocytes but not of resident macrophages. Human CD38 with functional activity has also been discovered in the outer membranes of red blood cells and platelets [13]. Within the B cell lineage, CD38 exhibits a unique distribution that is not seen very often in the immune system, characterized by elevated expression during early phases until the transitional states, followed by a continuous downregulation in the mature naïve and memory compartments, with the potential for re‐expression in certain processes, such as the germinal center reaction [5, 14, 15]. During the formation of ASCs, which represent the final stages in B cell development, CD38 reappears on the surface with high expression [16].

The participation of CD38 in shaping cellular responses depends on the cell type, developmental stage, activation state, and microenvironment [12, 14, 15, 17]. Based on the large amount of scientific evidence available on this protein, we may infer that its involvement in signaling depends significantly on its molecular surroundings on the cell surface, on the shared intracellular signaling routes, as well as on its enzymatic activity. This might be the underlying reason for the conflicting observations involving different cell types at various functional states, which lessens the surprise of even contradicting results about the contribution of CD38 in the immune system.

The complex, often paradoxical observations about its role in signaling in immune cells derive from the fact that this molecule has been studied in very distinct contexts: different cell types at different developmental stages from different model organisms [18–20], in health [14], and disease such as multiple myeloma (MM) [21], acute lymphoid and myeloid leukemia (ALL, AML) [22], chronic lymphocytic leukemia (CLL) [23, 24], and autoimmunity [25]. On one hand, CD38 is merely a marker to define subsets of immune cells, but on the other, it plays a significant role in the etiology of diseases, has immense potential to influence the microenvironment of tissues, and also functions as a prognostic marker [26].

The role of CD38 may vary across B cell groups based on their differentiation status. Given that intracellular Ca^2+^ response is a sensitive indicator of B cell function, in this work we attempt to better describe the effect of CD38 cell surface expression on the rapid Ca^2+^ flux response of different B cell subsets from healthy individuals using an adapted approach — which is unique in terms of accuracy and data output, highlighting the importance of well‐structured pipelines that are suitable for flow cytometry acquired kinetic measurements on intracellular Ca^2+^ flux. Immune cells in this study were assessed ex vivo, without culturing, in their most natural state possible, as considerable effort was invested in the preanalytical and analytical phases to create conditions with little to minimum impact on the cells.

## 2. Materials and Methods

### 2.1. Study Design and Sample Collection

Peripheral blood was collected from 15 healthy volunteers by venipuncture into six 10 mL lithium heparin tubes from each person. Peripheral blood mononuclear cells (PBMC) were isolated using Ficoll‐Paque (GE Healthcare, Chicago, IL, USA) gradient centrifugation, washed twice in phosphate buffered saline (PBS) then resuspended in RPMI 1640 containing 5% fetal bovine serum (FBS, Sigma–Aldrich, St. Louis, MO, USA).

Healthy volunteers were fully informed, and written consent was signed by each participant. The study was approved by the Regional Ethics Committee of the Medical Centre, University of Pécs in accordance with the Helsinki Declaration (No. RIKEB 5913/2015). The recruitment and measurements were conducted between January 6, 2020 and February 11, 2021.

### 2.2. Cell Surface Labeling

Isolated PBMCs were labeled with the following antibodies: CD3‐PerCP, CD19‐PE, CD27‐PE‐Cy7, IgD‐APC, and CD38‐APC‐Cy7. All fluorochrome‐conjugated antihuman antibodies were purchased from Sony Biotechnology (San Jose, CA, USA). CD markers were carefully titrated to 1 × 10^7^ cells in 100 µl labeling volume in RPMI. Cells were incubated for 30 min with the antibodies at room temperature in the dark, then washed with RPMI, centrifuged (400*g*, 7 min) and resuspended in RPMI for further processing. All the tubes were labeled separately and sequentially; measurements of each tube were carried out following a standardized time to let the possible effects of surface labeling subside.

Considering the large number of cells in a relatively low volume, 30 min incubation time allowed the antigen–antibody binding to approach maximal effective binding without stretching into long periods that could favor time‐dependent receptor degradation or severe stress to the cells, as tested at the titration phase before the main experiments. Maintaining the cells’ viability at the highest possible percentage was another crucial factor, because correct calcium flux measurements can only be performed on live, functioning cells.

Our method uses APC‐conjugated antihuman IgD clone IA6‐2, which is a full‐length mouse IgG2a κ monoclonal antibody with two identical Fab regions that can attach to two neighboring membrane‐bound IgD molecules on a single B cell. Local receptor dimerization may result from bivalent binding, but it does not cause large downstream Syk phosphorylation, PLCγ2 activation, complete ER calcium depletion, or fast actin‐dependent endocytosis. Recurrent multivalent crosslinking and signalosome spatial aggregation are needed for IgD signaling [17, 27]. BCR internalization is also highly temperature dependent. At 37°C, cross‐linked BCRs are internalized rapidly and cleared from the surface within 30–60 min. As the lipid bilayer is rigidified at 4°C, endocytosis is blocked and protein mobility is abolished. While endocytosis is not completely blocked at room temperature (22–24°C), the process is slowed sufficiently so that the loss of surface IgD is negligible during the 30 min period of the protocol [28–30].

### 2.3. Reconstitution of Fluo4 Acetoxymethyl‐Ester Dye

50 µg Fluo4 (F14201) was dissolved in 4.558 µl DMSO (10 mM stock solution). About 1 µl of the stock was added to 1 µl 20% (w/v) pluronic acid (5 mM solution). A total of 2 µl from the 5 mM solution was added to 98 µl RPMI (100 µM solution). To reach the 5 µM working concentration, 15 µl from the 100 µM solution was added to 285 µl of the surface‐labeled cells in RPMI.

### 2.4. Gating Strategy and Pre‐Processing

Compensation was carried out in FlowJo (v10.7.1., Becton, Dickinson & Company, NJ, USA) for each measurement day. Flow cytometry gating strategy included time gates in which flow stability was checked (y:FSC‐A/x:time, y:SSC‐A/x:time). Doublet discrimination was performed based on forward scatter height and area (y:FSC‐H/x:FSC‐A). Lymphocyte population was identified in a side scatter/forward scatter area (y:SSC‐A/x:FSC‐A) gate. In the kinetic measurements only the properly loaded cells with Fluo4 from the lymphocyte gate were included to the further analysis, except for the Fluo4 fluorescence minus one (FMO) controls. The same gating strategy was applied to the FMO controls as to the main samples, and the FMO controls were created from each individual’s own sample to precisely determine the CD38 boundary for each subpopulation and individual investigated. In regard of Fluo4 staining in this panel, the Fluo4‐FMO was used with the intent to exclude completely unloaded cells as a technical quality‐control step. At least 95% of all the PBMC was loaded successfully with Fluo4 in all tubes/measurements. CD3 served as a dump channel to exclude T cells, whereas B cells were identified by CD19 positivity. The B cell population that was selected in this manner was exported from the baseline and kinetic measurements into separate FCS files using FlowJo. Gates were set in FlowJo using the FMO controls as a reference for the negative boundary for each fluorophore.

In our phenotypic characterization setting, we defined the IgD^+^ CD27^−^ cells as the naïve and transitional compartment [31–35]. Subgrouping these cells by the expression of CD38 revealed (1) a high subpopulation, (2) a positive, and (3) a negative subpopulation in the IgD/CD38 gate. The latter two were discriminated using the CD38‐FMO controls for each individual and subpopulation, respectively. The CD38^high^ transitional cells do not separate from the naïve CD38^+^ cells so clearly thus, the most logical way of differentiating these cells was to define them as the highest 2.7%–6.6% (median: 4.935 (4.293; 5.240)) of the CD38 expressing cells in the naïve and transitional counterpart on the IgD/CD38 gate. The percentage data is based on our own experience by visually observing the distribution on the plots and on scientific literature [36, 37].

The CD19^+^ IgD^+^ CD27^+^ cells were defined as the unswitched memory population, the CD19^+^ IgD^-^ CD27^+^ cells as the CD27^+^ switched memory compartment, and double negative or late memory B cells were CD19^+^ IgD^-^ CD27^−^. Memory populations showed a bimodal expression of CD38 thus, a CD38 positive and a negative subpopulation was characterized in the unswitched, CD27^+^ and CD27^−^ memory gate with the help of the CD38‐FMO controls, while the antibody secreting cells (ASC) were identified as the CD19^+^ IgD^-^ CD27^high^ CD38^high^ cells in the CD27^+^ switched memory compartment (Figure S1A,B).

The PerCP antihuman CD3 (clone SK7), PE antihuman CD19 (clone SJ25C1), APC antihuman IgD (clone IA6‐2), and the APC/Cy7 antihuman CD38 (clone HIT2) antibodies were purchased from Sony Biotechnology, the PE/Cy7 antihuman CD27 (clone M‐T271) antibody from BioLegend.

### 2.5. B Cell Activation and Ca^2+^ Flux Measurements by Flow Cytometry

Titration series of the activating agents were performed in three biological replicates (three different individuals). B cells were activated (1) using an AffiniPure F(ab’)_2_ fragment goat antihuman IgG (F(ab’)_2_ fragment specific, cat# 109‐005‐097) at the concentrations of 0.5, 1, 10, and 20 µg/mL; (2) an AffiniPure F(ab’)_2_ fragment goat antihuman IgM (Fc5μ fragment specific, cat# 109‐006‐129) at the concentrations of 0.5, 1, 10, and 20 µg/mL from Jackson ImmunoResearch (Cambridgeshire, UK); and (3) ionomycin at the concentrations of 0.01, 0.1, 1, and 2 µg/mL (Sigma–Aldrich, St. Louis, MO, USA). We also measured a series of kinetic measurements without any kind of activating agent to check fluorescence intensity deviations originating from the instrumental setup.

The optimal working concentrations for the activating reagents were set based on the titrations as follows: 1 µg/mL for ionomycin, 10 µg/mL for anti‐IgG, and anti‐IgM. The necessary number of replications was decided as 15 by a power analysis performed on the AUC and maximum parameters comparing the main B cell compartments (Figure S2A,B).

Following cell surface labeling, washing, and re‐suspending in 285 µl RPMI, 15 µl Fluo4 dye from the 100 µM solution was added to the cells and incubated for 15 min at room temperature in dark. After washing and centrifugation, cells were suspended in 185 µl RPMI, which was supplemented with CaCl_2_ to reach the 1.8 mM Ca^2+^ concentration, and were left in the dark to allow the dye to de‐esterificate for 15 min. In the kinetic measurements on a BD FACS Canto II flow cytometer (Becton, Dickinson and Company, Franklin Lakes, NJ, USA) 60 s unstimulated baseline and 720 s activation phase was recorded from each tube. Figure S1D illustrates that the fluorescence intensity of the surface markers remained consistent throughout the kinetic acquisition period.

### 2.6. Data Generation From the Kinetic Analysis

Data of the gated populations (baseline and kinetic files separately) were imported to FacsKin; time shift between the baseline and kinetic file was entered, and the activation curve was generated using the median fluorescence intensity (MFI) values of the detected events. All curves were standardized to end at 780 s. During the analysis of immunoglobulin‐based activating agents and ionomycin, the optimal function to fit the data was the double logistic (Figure S1C). Nonactivated tubes were analyzed accordingly.

The mathematical description of the functions includes the following parameters: Starting value (can be standardized to 1 to allow comparison of different subsets and measurement days). Maximum value shows the maximum MFI of Fluo4 which correlates with the maximum level of cytoplasmic free Ca^2+^ reached during the activation. Ending value is the Fluo4 MFI at the end of the activation curve at 780 s, which represents the cytoplasmic Ca^2+^ level at the end of the observation period, in this experimental setup it is analogous with the plateau phase of the function. Time to reach maximum is the timepoint when the function reaches the maximum Fluo4 MFI, interpreted as the time the cells require to mobilize the maximum amount of ionized intracellular Ca^2+^ during the activation. Time parameters are representing the response capability of the cells to a certain stimulus on the time scale. Time to reach the 1^st^ 50% value is the time required to reach the half of the maximum Fluo4 MFI, time from 1^st^ 50% value to maximum is the time elapsed from the 1^st^ 50% Fluo4 MFI value to the maximum Fluo4 intensity, time from maximum to the 2^nd^ 50% is the time elapsed from the maximum to the 2^nd^ half of the maximum Fluo4 intensity in the descending phase. Slope at 1^st^ 50% describes how much the fluorescence intensity changes in 1 s in the ascending phase of the activation, indicating how quickly cells respond to the stimulus. Slope at 2^nd^ 50% value describes how much the fluorescence intensity changes in 1 s in the descending phase of the activation indicating how quickly the Ca^2+^ level decreases after the peak of the activation following a certain stimulus. The area under the curve (AUC) correlates with the total capacity of the cells to mobilize Ca^2+^ ions via a certain activation pathway (Figure S1C).

### 2.7. Controls and Reproducibility

Compensation was carried out using BD Calibrate 3‐color kit (Becton, Dickinson and Company, Franklin Lakes, NJ, USA) for PE and APC, anti‐mouse Ig, κ and Negative Control Compensation Particles Set (Becton, Dickinson and Company, Franklin Lakes, NJ, USA) for BV421, PE‐Cy7 and APC‐Cy7 conjugated antibodies, and the test subjects’ PBMCs for Fluo4 and the viability dye. At each measurement day for each activating agent, a sample without the addition of Fluo4 was measured in a kinetic manner for the same time as the test samples, which served as the Fluo4 FMO. Referring to the results from this tube we could exclude any alterations in the background fluorescence due to the activation process and allowed us to make sure the signal we see in the fluorescein isothiocyanate (FITC) channel, following the exclusion of the spread and spillover in the detector, comes from the Fluo4 intensity changes and only from it. Viability was checked at three different points of the experiments; (1) in a separate tube following the counting of the separated PBMC, using Zombie NIR viability dye (BioLegend, San Diego, CA) to assess the effects of the preanalytical workflow, (2) during the measurement of the fully labeled samples where only those cells were included into the analysis which were intact thus, could be loaded with Fluo4 up to an acceptable intensity—except for the Fluo4 FMO controls where it is not applicable, and (3) at the end of the kinetic phase of the measurements where trypan blue was added to the tubes to check viability in a Bürker chamber to make sure no rapid cell death occurred. Comparability of the experiments (different individuals and distinct subpopulations) was ensured by the standardization offered by the FacsKin software/package during which the baseline measurements were standardized to 1 in order to assess and interpret the kinetic phase accordingly even from different basal Fluo4 intensity levels.

The responses of the CD27^−^IgD^−^/CD27^+^IgD^−^ compartments to anti‐IgM, and of the CD27^−^IgD^+^/CD27^+^IgD^+^ populations to anti‐IgG were used as an internal negative control (Figure S3A–D). The evaluated patterns closely resembled those of the nonactivated cells, demonstrating that the gating effectively separated the compartments, and that the anti‐IgM antibody did not trigger Ca^2+^ signaling in IgG expressing cells, and the anti‐IgG antibody did not trigger substantial Ca^2+^ flux in the IgM BCR bearing cells.

### 2.8. Analysis and Statistics

Distribution was checked using the D’Agostino–Pearson, Anderson‐Darling, Shapiro–Wilk, and Kolmogorov–Smirnov tests on all the datasets and statistical probes were selected accordingly. Dose–response curves were fitted with nonlinear regression, outliers were identified and excluded by the ROUT method (*Q* = 1%). Population prevalence, fluorescence intensity data, and parameters from the main kinetic measurements were processed using Mann–Whitney test or Kruskal–Wallis nonparametric ANOVA with Dunn’s post hoc test. The level of significance was set at 0.05. Outliers were identified and excluded by the ROUT method (*Q* = 8%). Analysis and graphing were carried out in GraphPad Prism 10 (GraphPad Software, San Diego, CA, USA). Dose–response characteristics of the circulating B cell subpopulations upon anti‐IgM and anti‐IgG stimulation were assessed by nonlinear curve fitting, agonist vs response (three parameters), using the concentrations of the anti‐Ig antibodies and the maximum values of the kinetic measurements as response, to calculate the half maximal effect concentrations (EC_50_).

The clustered heatmap and the scatterplot were created in Python (v. 3.11.4) by using the numpy (v 1.24.4), pandas (v. 2.0.3), matplotlib (v. 3.7.2), and seaborn (v. 0.13.2) packages. To compare the kinetic profile of different immune cell subpopulations we used the clustermap function of seaborn on the measured parameters (such as basal Ca^2+^ activity, EC_50_ for maximum, maximum, AUC, and ending) which provides a visual representation of similarities and differences in their patterns. The clustermap function performs a hierarchical clustering of the data, for which the distance between clusters was calculated by the average linkage method using Euclidean distance as a metric. The examined parameters are situated in highly distinct ranges, therefore the transformation of this data before clustering was necessary to be present on the same scale. For this purpose, the StandardScaler method of scikit‐learn (v. 1.3.0) was utilized, which standardizes the data by removing the mean and by scaling to unit variance. The heatmap displays these transformed values.

In the scatterplot, data points are indicated in three different colors according to a specific grouping. These groups were identified by K‐means clustering that was performed on two parameters: the basal Ca^2+^ activity and the calculated ratio of EC_50_ for maximum/maximum value. In this case, the data was scaled with the same StandardScaler method and the settings for K‐means clustering (scikit‐learn) were as follows: the number of clusters was three, the number of times the algorithm was executed with different centroid seeds was 10, the maximum number of iterations for a single run was 300 and the model used the k‐means++ method for initialization. Two metrics, inertia and silhouette score, were examined to choose an optimal number of clusters.

Final figures were constructed, and their size and resolution adjustments were made in Procreate (Savage Interactive Pty Ltd, v. 5.3.7). Featured image was created in BioRender (Toronto, Canada). We did not use any kind of artificial intelligence (AI) to help generate content, write code, or process data. The code for the computational analysis can be found at the following GitHub link: https://github.com/peterkaltenecker/CD38_in_B_cell_activation.

## 3. Results

### 3.1. Frequency and Basal Calcium Activity of B Cells Categorized by CD38 Expression in Each Peripheral Subset

#### 3.1.1. CD38^high^ Transitional B Cells Had Higher Basal Calcium Activity Than the More Mature Transitional and Naïve Cells

**Figure 1:**
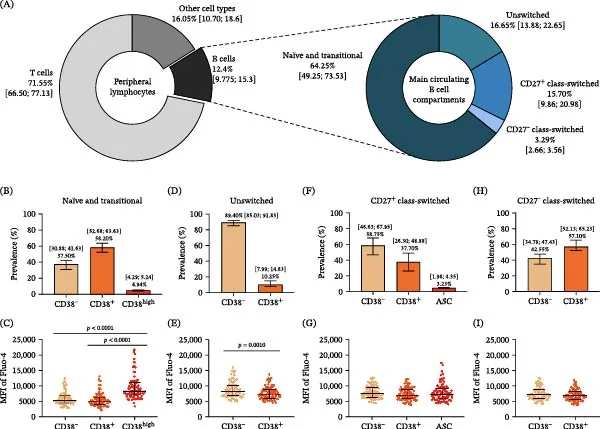
Prevalence and basal Ca^2+^ activity of circulating B cell subsets. (A) Frequency of the naïve and transitional, unswitched memory, and class‐switched memory cells in the circulation of healthy individuals categorized by CD38 expression. Median and IQR. (B, D, F, H) Prevalence of the subpopulations (C, E, G, I) Basal Ca^2+^ activity (MFI of Fluo‐4) of the circulating B cell compartments. Data points represent all the measurements made on the samples of 15 healthy individuals, which equals with the number of individuals x number of activations (anti‐IgM, anti‐IgG, ionomycin, nonactivated control)x2 (because the baseline and kinetic files were analyzed separately in this section). Mann–Whitney test and Kruskal–Wallis nonparametric ANOVA with Dunn’s post hoc test, *a* = 0.05.

The naïve and transitional compartment represented the 64.25% [49.25; 73.53] of total B cells in healthy individuals (Figure 1A). Dividing this compartment by CD38 expression, CD38^−^, CD38^+^, and CD38^high^ populations could be revealed by applying the most accurate, FMO–based manual gating method possible in our experimental setup. The prevalence of these three populations within the compartment turned out to be as follows: CD38^+^ (58.20% [52.68; 63.63]) >CD38^−^ (37.50% [30.88; 41.63]) >CD38^high^ (4.94% [4.29; 5.24]) (Figure 1B). The MFI of the Fluo4 dye was significantly higher in the CD38^high^ population compared to the CD38^−^ (*p* < 0.0001) and to the CD38^+^ (*p* < 0.0001) subsets, suggesting that transitional cells first entering the blood stream have higher basal Ca^2+^ activity compared to the rest of the naïve and transitional compartment (Figure 1C).

#### 3.1.2. The Majority of the Unswitched Memory B Cells Were Negative for CD38 and Had Higher Basal Ca^2+^ Levels Compared to the Positive Counterpart

The unswitched compartment represented the 16.65% [13.88; 22.65] of total B cells in healthy individuals (Figure 1A). The prevalence of CD38^−^ and CD38^+^ populations within the compartment turned out to be as follows: CD38^−^ (89.40% [85.03; 91.83]) >CD38^+^ (10.25% [7.99; 14.83]; Figure 1D). The basal ionized Ca^2+^ concentration was significantly higher in the CD38^−^ population compared to the CD38^+^ cells (*p* < 0.0010; Figure 1E).

#### 3.1.3. The CD27^+^ and CD27^−^ Switched Memory Cells, Together With the ASC Populations, Displayed No Major Variations in Their Basal Ca^2+^ Activity

The CD27^+^ class‐switched memory compartment represented 15.70% [9.86; 20.98] of total B cells in healthy individuals (Figure 1A). Dividing this compartment by CD38 expression, CD38^−^, CD38^+^, and CD38^high^ populations could be found, where the CD38^high^ population represented the circulating ASCs. The prevalence of these three populations within the compartment turned out to be as follows: CD38^−^ (58.75% [46.63; 67.95]) >CD38^+^ (37.70% [26.30; 48.88]) >CD38^high^ (3.23% [1.98; 4.35]; Figure 1F). There was no significant difference comparing the basal MFI of the Fluo4 dye among these populations (Figure 1G).

The CD27^−^ switched memory compartment represented 3.29% [2.66;3.56] of total B cells in healthy individuals (Figure 1A). The prevalence of CD38^−^ and CD38^+^ populations within the compartment was: CD38^+^ (57.10% [52.13; 65.23]) > CD38^−^ (42.55% [34.78; 47.43]) (Figure 1H). No significant difference could be found comparing the basal MFI of the Fluo4 dye between these populations (Figure 1I).

### 3.2. Naïve and Transitional Compartment

#### 3.2.1. CD38^high^ Cells in the Naïve and Transitional Compartment Were More Responsive to anti‐IgM Stimulation Than the Negative and Positive Subpopulations

**Figure 2:**
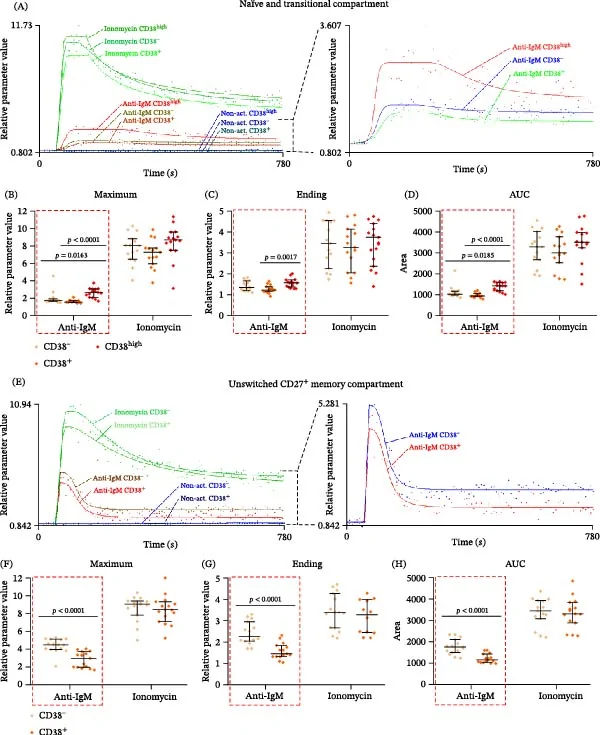
Kinetic properties of the Ca^2+^ flux in the naïve and transitional, and unswitched B cell compartments, divided by the CD38 surface marker, on the periphery of healthy individuals. (A, E) Representative Ca^2+^ kinetic curves generated in response to anti‐IgM, and ionomycin stimulation together with the non‐activated curve. Colors are not matched between the inset and the overview, the curves should be identified by their labels. (B, F) The maximum relative parameter value, representing the maximum Ca^2+^ concentration. (C, G) The ending value, analog with the Ca^2+^ concentration at the plateau phase. (D, H) The AUC value, as the cells’ total capacity to mobilize Ca^2+^ ions, plotted against the activating agents in the CD38^−/+/high^ subpopulations. Mann–Whitney test and Kruskal–Wallis nonparametric ANOVA with Dunn’s post hoc test, *a* = 0.05, *n* = 15.

The CD38^high^ subpopulation reached significantly higher maximum Ca^2+^ concentration during anti‐IgM stimulation and had higher total capacity to mobilize Ca^2+^ ions into the cytoplasm compared to the CD38^+^ and CD38^−^ cells, while the only notable difference in Ca^2+^ concentration during the plateau phase was observed between the CD38^+^ and CD38^high^ cells (Figure 2A–D).

The CD38^high^ cells expressing IgD and IgM demonstrated a greater susceptibility to stimulation; their peak and overall capacity to mobilize Ca^2+^ into the plasma exceeded that of the other two counterparts that required 1.511 and 2.153 times more stimulation, respectively, than the CD38^high^ cells to achieve their half maximal effect, with their basal Ca^2+^ activity being ~1.6 and 1.5 times lower. Despite the absence of a significant difference in maximum values between the CD38^+^ and CD38^−^ cells, the former required a 1.4‐fold higher amount of anti‐IgM to reach a comparable maximum Ca^2+^ concentration (Figure S4A,B).

The CD38^high^ transitional subpopulation also showed a slower decrease of free Ca^2+^ after reaching the peak concentration as observed in the temporal dynamics compared to the negative counterpart, and in the slope characteristics compared to the positive cells, while the other time and slope parameters did not differ significantly within the compartment (Figure S5A–F). As expected, ionomycin, serving as a positive control for Ca^2+^ mobilization, induced a quicker increase and subsequent removal of Ca^2+^ ions from the cytoplasm compared to anti‐IgM stimulation (Figure S5E,F).

### 3.3. Unswitched Memory B Cells

#### 3.3.1. CD38^−^ Unswitched Memory B Cells Were More Responsive to Anti‐IgM Stimulation Than Their Positive Counterpart

Dividing the unswitched compartment according to the expression of the CD38 molecule, a remarkably different activation pattern could be revealed in the CD38^−^ and CD38^+^ subpopulations. IgM activation resulted in elevated maximum, ending, and AUC values in the CD38^−^ subpopulation compared to the positive cells, but such differences were not seen after ionomycin stimulation (Figure 2E–H). Notably, while the peak value in the CD38^−^ subpopulation was 1.55 times higher, the cells required substantially more (2.9 times) anti‐Ig activating agent to reach the half maximal effect than their positive counterpart, exhibiting a reduced susceptibility to anti‐IgM stimulation (Figure S4A,B).

The evoked Ca^2+^ signal to anti‐IgM addition in the CD38^−^ cells required significantly less time to reach the maximum, to reach the 1^st^ 50%, and time from 1^st^ 50% to maximum concentration compared to the CD38^+^ cells, while we did not observe any differences in the descending phase on the time axis between the subpopulations (Figure S5G–J). The difference in rapidity is also seen in the ascending slope parameter on Figure S5K without a significant difference in the descending phase (Figure S5L).

Despite having comparable basal Ca^2+^ activity to the transitional CD38^high^ cells, the peak of the CD38^−^ unswitched subpopulation was 1.68 times higher. The total capacity (AUC) of these cells to mobilize Ca^2+^ surpassed the average of all other subpopulations by 49.55%, and their activation generated the highest plateau phase recorded during the experiments.

### 3.4. CD27^+^ Switched Memory B Cells

#### 3.4.1. Response Capability and Rapidity Were in Inverse Relation With the Level of CD38 Expression in the CD27^+^ Switched Memory Compartment

**Figure 3:**
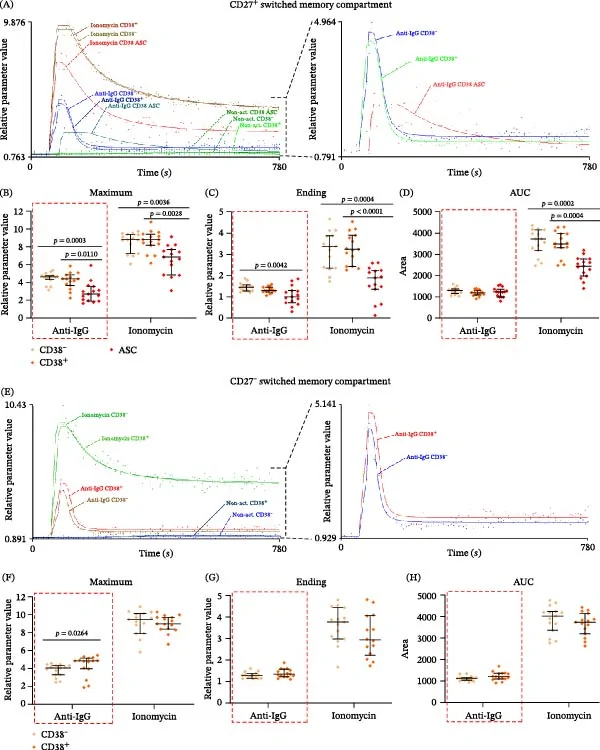
Kinetic properties of the Ca^2+^ flux in the CD27^+^ switched and CD27^−^ memory B cell compartment, divided by the CD38 surface marker, on the periphery of healthy individuals. (A, E) Representative Ca^2+^ kinetic curves generated in response to anti‐IgG, and ionomycin stimulation together with the non‐activated curve. Colors are not matched between the inset and the overview, the curves should be identified by their labels. (B, F) The maximum relative parameter value, representing the maximum Ca^2+^ concentration. (C, G) The ending value, analogous to the Ca^2+^ concentration at the plateau phase. (D, H) The AUC value, as the cells’ total capacity to mobilize Ca^2+^ ions, plotted against the activating agents in the CD38^−/+/high^ subpopulations. Mann–Whitney test and Kruskal–Wallis nonparametric ANOVA with Dunn’s post hoc test, *a* = 0.05, *n* = 15.

CD38^−^ and CD38^+^ cells did not show significant difference in their maximum, ending and AUC Ca^2+^ concentrations, although a tendency is present, which suggests that the responsivity of CD38^−^ cells is higher compared to the CD38 expressing cells. This is also supported by the dose–response statistics showing that CD27^+^ CD38^−^ cells responded already to a lower concentration of anti‐IgG (Figure S4C,D). High CD38 expression in ASCs is linked to lower responsivity upon stimulation with anti‐IgG, and ionomycin as well, compared to the CD38^−^ and CD38^+^ subpopulations (Figure 3A–C). Interestingly this tendency did not appear so strikingly in the AUC values following anti‐IgG stimulation (Figure 3D). ASCs were also slower to respond and required more time to go through the entire Ca^2+^ flux compared to the class‐switched CD38^−^ and CD38^+^ cells (Figure S6A–F). These cells required the lowest amount of activating agent to produce an elevation in their free Ca^2+^ concentration (Figure S4C,D), and the attained peak was 1.44 times lower than the average of the IgG‐BCR bearing class‐switched memory B cells (including both CD27^+^ and CD27^−^ cells; Figure 3A,B). The ASCs also showed the lowest plateau phase among all the examined subpopulations (Figure 3C).

### 3.5. CD27^−^ Double Negative Switched Memory B Cells

#### 3.5.1. CD38^+^ and CD38^−^ Cells did not Differ Remarkably in Their Ca^2+^ Response Characteristics in the CD27^−^ Memory Compartment

The CD38^+^ cells attained an elevated peak of intracellular Ca^2+^concentration in this compartment, although exhibited no difference in the ending, and AUC values compared to the negative cells (Figure 3E–H). The Ca^2+^ flux did not show remarkable differences in the time and slope parameters either between the CD38^−^ and CD38^+^ subpopulations (Figure S6G–L), while the CD38^+^ cells required 2.44 times lower amount of activating agent to achieve a maximum 1.15 times greater than that of the CD38^−^ cells (Figure S4C,D).

**Figure 4:**
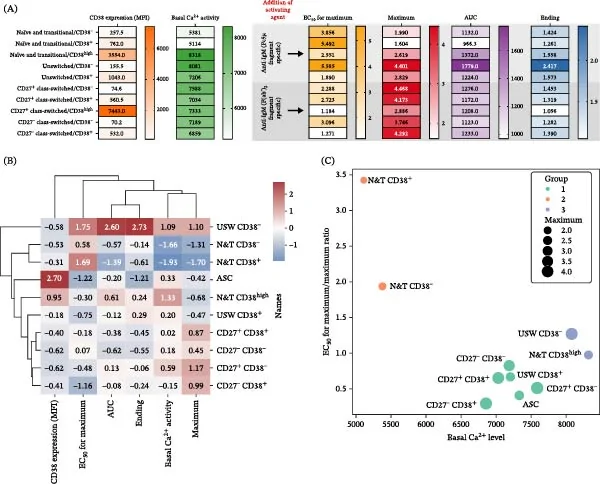
A cumulative depiction of results encompassing the basal Ca^2+^ activity from baseline measurements, the half maximal effect concentrations (EC_50_) calculated from the maximum values, as well as the maximum, AUC, and ending values from the kinetic measurements. (A) Tabular view of raw data. (B) Heat map showing hierarchical categorization of subpopulations and variables after dataset scaling. (C) B cell subsets defined by CD38 expression visualized according to basal Ca^2+^ levels and the EC_50_:maximum value ratio. Dot size indicates the reached maximum Ca^2+^ concentration of each subset. Dot color labels the grouping of the subsets by K‐means clustering.

The findings of Sections 2 to 5 of the Results are summarized in Figure 4A in a tabular manner.

### 3.6. Summary of Findings Supported by Additional Analytical Methods

#### 3.6.1. Hierarchical Cluster Mapping of CD38 Expression and Ca^2+^ Homeostasis‐Related Kinetic Parameters Highlights Similarities and Differences Between B Cell Subpopulations

Figure 4B illustrates an interpretation of the relationships across subpopulations formed based on the similarity in the pattern of variables within each subpopulation gated based on the CD38 expression in each circulating B cell compartment. Although raw values of the parameters fall into distinct ranges, this problem was solved by standardizing the data to the same scale (Figure 4B shows these modified values). According to this pattern, we observed that the class‐switched CD27^−^ and CD27^+^ memory cells are in proximity to each other, irrespective of their CD38 expression levels, and that these cell populations differ greatly from ASCs. This approach also confirmed the uniqueness of the unswitched CD38^−^ cells regarding their Ca^2+^ flux characteristics and highlighted the substantial contrast between this group and its CD38^+^ counterpart. In addition, the transitional CD38^high^ subpopulation was indeed highly different from the CD38^+^ and CD38^−^ naïve and transitional cells.

#### 3.6.2. Introducing the EC_50_: Maximum Ratio as an Independent Parameter for Activation Capability

Another interpretation of these differences is the representation of the subpopulations based on their basal Ca^2+^ levels and the EC50:maximum value ratio; the latter indicating the required activating stimulus for a one‐unit increase during the elevating phase to achieve the maximal response to a particular activating agent. In Figure 4C we see an unequivocal compartmentalization of the subpopulations that is similar to the results of the hierarchical clustering in Figure 4B but performed on the EC_50_:maximum ratio as an independent parameter and on the basal Ca^2+^ activity using K‐means clustering. This context reveals three separate clusters. The CD27^−^ and CD27^+^ class‐switched cells are grouped with the unswitched CD38^+^ subpopulation, exhibiting nearly similar baseline activity and activation capability (labeled green). Naïve and transitional CD38^−^ and CD38^+^ cells (depicted in orange) are positioned distantly from the other two clusters, exhibit reduced basal Ca^2+^ levels, require a stronger stimulus, and ultimately attain a comparatively moderate maximum Ca^2+^ level in response to anti‐Ig activation. The unswitched CD38^−^ cells and the transitional CD38^high^ cells display similar patterns in the examined parameters, forming the third, rather cohesive cluster (purple) with the highest baseline Ca^2+^ level among the subpopulations and an intermediate activation capability.

## 4. Discussion

CD38 plays a fundamental role in physiology by modulating immunometabolic homeostasis, inflammation, and autoimmune responses. To fully understand how the expression and engagement of CD38 affects different types of B cells when activated through the BCR requires a scrutiny of results from a wider viewpoint, considering other functional assessments in addition to our data on kinetic flow cytometric measurements. While our research could not fully separate the functional effects of CD38, in this study we found some interesting details regarding the differences in kinetic Ca^2+^ responses of each circulating B cell compartment after grouping the cells by their CD38 expression.

We examined the induced Ca^2+^ flux after BCR engagement in peripheral B cell populations with negative, positive, and high CD38 expression levels, obtained from healthy individuals, employing a method that adjusts the functions to fit the varying fluorescence intensity of a Ca^2+^ sensitive dye recorded over 10 min. Anti‐Ig activating agents were titrated to a concentration at which all the subpopulations had a measurable response, evidenced by a rise of intracellular Ca^2+^ in a double logistic manner, characterized by a peak and a plateau phase.

In our experimental setting, B cells with IgM‐type BCRs behaved differently from the IgG bearing cells and displayed an even greater intracompartmental variability in their Ca^2+^ kinetic characteristics in accordance with their respective expression levels of CD38. On the other hand, IgG^+^ class‐switched memory cells were categorized rather uniformly based on their activation patterns, demonstrating robust responses to anti‐IgG stimulation, without any significant correlations to their CD38 expression levels. IgM and IgG expressing B cells participate in separate stages of the immune response, with IgM being crucial for the initial reactions and IgG supplying long‐term, high‐affinity immunity following class‐switching [38]. Accordingly, the processes in which CD38 is involved when expressed on the cell surface, as well as its role during cellular activation, might differ in these two types of cells. Moreover, in line with previous findings [39] we could see important differences in the pattern of the flux between the IgM expressing naïve and transitional cells and the unswitched IgM memory compartment, suggesting that aside from the BCR isotype, the maturation state of B cells provides a platform as well to a differential regulation in which the CD38 molecule is also variably implicated [40]. Possible explanations for this may be the composition of inhibitory and activating receptors on the cells, and consequently, the spatial arrangement and lateral associations on the surface [41], and how these are changing during maturation and differentiation. For instance, as naïve B cells transform into memory cells and go through class switching, CD40 expression increases while CD5 and the leukocyte‐associated immunoglobulin‐like receptor‐1 (LAIR‐1) get downregulated [39].

Approaching the contribution of CD38 in shaping Ca^2+^ flux in B cells, it is essential to focus on the specific cell surface environment in which it is expressed. Studies in leukemia and lymphoma cells suggest that CD38 is a component of the BCR co‐receptor family, as indicated by its co‐localization with members of the BCR co‐receptor complex, including Igα, Igβ, CD81 [42], and CD21 [43]. Furthermore, in tonsillar B cells and continuous lines, the lateral interaction with CD19, the principal activating co‐receptor for B lymphocytes, is essential for the signaling capabilities of CD38. The inhibition of CD19 prevented CD38 from interfering with Ca^2+^ fluxes and from relocalizing to the CD19:CD81 complex [44].

Recent findings indicate that targeting CD38 with daratumumab impedes the lateral interaction of IgM and CD19, proposing that CD38 is necessary to IgM:CD19 synapse formation and could regulate the activation threshold of naïve and resting B cells by affecting the localization of CD19 [41]. Molecular complexes including CD38, CD49d, matrix metalloproteinase‐9 (MMP9) [9], and CD29 have been identified in CLL associated with a less favorable outcome [45]. Reports also demonstrate that therapeutic anti‐CD38 antibodies have diverse effects on B cell signaling, highlighting complexities involved in the pathways influenced by CD38 engagement [46, 47].

The reported interactions vary based on the specific lineage and also change according to the developmental stages of the cells. These co‐operations proved to be essential for the signal transduction of CD38, but not for its enzymatic activity [44]. The findings so far demonstrate that given the very short intracellular domain of this molecule, CD38 presumably requires the use of the intracellular signal transduction machinery associated to the BCR complex to be able to transmit or shape a signal [44], and that it has the potential to access the BCR signaling pathway if physically co‐capped with the complex shown in murine B cells [48]. The rationale for this is likely rooted in a compelling nano‐evolutionary event roughly 500 million years ago in jawed fish, when lymphocytes with somatic recombination‐derived antigen receptors developed, TCR and BCR emerged [49], CD38 acquired the ability to employ their signaling routes. This way, CD38 gained the possibility to shape the signals coming through receptor complexes or guide their own into the cell with the help of the shared pathways [50].

On the other hand, IgM and IgD feature cytoplasmic domains containing three amino acids, while IgG, IgA, and IgE isotypes have intracellular domains of 25–30 amino acids, facilitating a faster and enhanced response by offering binding sites for a broader array of components [51]. In addition to immunoglobulin class‐switching, a comprehensive reconstruction of signaling routes occurs regarding how the cells integrate signals and regulate transcription. IgG, unlike IgM, interacts with signaling molecules such as growth factor receptor‐bound protein 2 (Grb2) and synapse‐associated protein 97 (SAP97) that propagate the formation of immunological synapse and downstream signaling, including Ca^2+^ mobilization [52, 53]. Class‐switched cells have considerably higher levels of stromal interaction molecule 1 (STIM1) and inositol 1,4,5‐trisphosphate receptor (IP3R) as well in comparison to naïve cells [39]. Previous findings about how the sustained oscillations at the ending phase of the Ca^2+^ flux after BCR engagement leads to a maintained ERK1/2 phosphorylation [39] is putting our results in context. The measure of overall capacity of IgG‐expressing memory B cells to mobilize Ca^2+^ into the cytoplasm relied slightly more on the plateau Ca^2+^ levels than on the maximum value when compared to the IgM^+^ naïve and memory cells.

The role of CD38 in influencing Ca^2+^ signaling and subsequent cellular responses within the diverse physical and signaling contexts of various B cell types requires further explanation. Concerning its function in circulating B cells, one role is definitively established: CD38 expression is induced on naive B lymphocytes upon activation, increases prior to germinal center entry, peaks during entry, subsequently declines throughout centrocyte and centroblast development, and is markedly expressed on ASCs [15, 54]. In both cases of elevated expression, cells are at phases requiring the ability to penetrate tissues and engage with other cells. Indeed, CD31/PECAM‐1, the nonsubstrate ligand for CD38, primarily controls leukocyte adherence to endothelial cells, hence enabling movement and cellular interactions [12]. It eventually became evident that the natural ligands of CD38 cannot account for the diverse effects induced in various cell types by anti‐CD38 monoclonal antibodies [55]. The cellular outcomes of surface receptor ligation are determined by the ligand‐receptor affinity and avidity, the binding characteristics, and resulting alterations in signal intensity and duration. The threshold effect concerning ligand concentration must also be considered [56]. While ligation of CD38 on B cell precursors from the bone marrow and in derived lines caused growth suppression and apoptosis [57], in circulating mature B cells it triggered anti‐apoptotic, and proliferative pathways and stimulated the production of cytokines, such as IL‐6, GM‐CSF, and IFN‐γ [14].

Regarding the striking differences in the Ca^2+^ flux parameters found between the CD38^−^ and CD38^+^ unswitched cells, we can speculate that if CD38 positivity may indicate a state potentially signifying a commitment to differentiation or entry into the GC [5, 14, 15], it possibly has a role in shaping the cellular metabolic processes as well [11]. A transition from a quiescent to an active state involves the redistribution of nutrients across different processing routes [58]. Since CD38 is the primary NAD^+^ hydrolase in mammalian cells, and NAD^+^ serves as a crucial co‐factor in cellular metabolism by transporting high‐energy electrons throughout metabolic pathways, it makes sense to hypothesize its involvement in metabolic regulation, despite the topological dilemma that arises from the presence of an external catalytic site on the cell surface [4]. Moreover, the byproducts of this enzymatic activity are secondary messengers capable of mobilizing Ca^2+^ ions from intracellular reserves; hence, the participation of CD38 in cellular activation is assumed to induce changes in Ca^2+^ regulation and its consequences in immune cells [5, 15].

Numerous studies in the ‘90 s and ‘00 s independently identified that CD38 engagement on different immune cell types resulted in the tyrosine phosphorylation of the c‐Casitas B‐lineage lymphoma (c‐cbl) proto‐oncogene [57, 59–63]. Following the initiation of BCR signaling, Src‐family kinases phosphorylate the immunoreceptor tyrosine‐based activation motifs (ITAMs) present on CD79a and CD79b. The ITAMs serve as a docking site for spleen tyrosine kinase (Syk), which modulates actin dynamics to enable the internalization and processing of the antigen‐loaded BCR complex [64]. Internalized, loaded BCR complexes interact with the major histocompatibility complex II (MHC II) via a ubiquitin‐dependent mechanism [65]. C‐Cbl, an E3 ubiquitin ligase family member, actively engages in antigen‐driven BCR ubiquitination and facilitates BCR‐mediated antigen processing, thereby orchestrating antigen‐induced BCR signaling and ubiquitination, as well as the fast processing and presentation of cognate antigen to augment the development of the humoral immune response [66].

In light of c‐cbl serving as a functional connection point between the relevant processes [66], and that CD38 seems to be necessary for the interaction of the membrane‐bound immunoglobulins and CD19 [41], this molecule may contribute to the precise regulation of antigen receptor signaling and acts as a regulator of the B cell activation threshold. This fine‐tuning, among other factors, might account for the differences in the CD38^−/+/high^ cells that manifest as measurable variations in the Ca^2+^ flux patterns.

## 5. Conclusions

The observed differences in Ca^2+^ flux characteristics among circulating B cell subpopulations likely stem from factors beyond the expression of the CD38 molecule, although it may play a contributory role in differentiation processes and functional determination, depending on the responsive elements in its regulatory region. Furthermore, the presence of the functioning CD38 protein as an enzyme and co‐receptor on the cell membrane is likely to affect the Ca^2+^ homeostasis and signaling in B cells. Due to the distinct surface protein composition and maturation stage of B cell subpopulations, CD38 forms lateral associations with different kinds of surface molecules on each cell type, leading to varied outcomes. With our experiments we could not assign a universal role to CD38 regarding Ca^2+^ signaling throughout the circulating B cell compartments; however, we identified an association between intracompartmental variability in the Ca^2+^ flux patterns and CD38 expression, which was pronounced in populations expressing an IgM‐type BCR.

## Funding

The research was financed by the Thematic Excellence Program 2021 Health and National Defense, the National Security Sub‐Programs of the Ministry for Innovation and Technology in Hungary, within the framework of the EGA‐10 and by the Project No. RRF‐2.3.1‐21‐2022‐00012, titled the National Laboratory on Human Reproduction that has been implemented with the support provided by the Recovery and Resilience Facility of the European Union within the framework of Program Széchenyi Plan Plus and by the University of Pécs, Medical School.

## Conflicts of Interest

The authors declare no conflicts of interest.

## Supporting Information

Additional supporting information can be found online in the Supporting Information section.

## Supporting information


**Supporting Information** Figure S1: Gating pipeline. (A) Flow cytometric gating strategy to define the main circulating B cell compartments. (B) FMO–based definition of CD38 expression in the main circulating B cell compartments. The full gating strategy, including the CD38‐FMO boundaries, was applied per individual and per subpopulation, creating a compartment‐specific threshold for CD38 positivity. The negative/positive gate boundaries were based on the resolution of negative populations and not set by chance or by a biased subjective approach. Contour plots were drawn in FlowJo using equal probability contouring, using 5% threshold. At 5% probability there are no outliers outside the lowest contour line in the “CD27^−^ class‐switched memory” population, thus, there is no error in picturing the events and the representation is consistent by using the same 5% threshold in all the plots. (C) Double logistic representation of intracellular Ca^2+^ flux during B cell activation. (D) Visual representation of marker stability by depicting the x/Time, y/fluorophore axes. The kinetic files were plotted as follows: x/Time, y/FSC‐A. Two identical sections were selected at 200 and at 600 s. The fluorescence intensity of the B cell markers was exported of these two gates. Median intensity of the B cell markers in a kinetic measurement from the 200 and 600 s gates. Figure S2: Power analysis. Unpaired measurements comparing the main B cell compartments. Calculated effect sizes (*d*) and sample sizes (*n*) for the possible combinations of B cell subpopulations, in the case of maximum and AUC values, upon anti‐IgM (A) and anti‐IgG (B) activation. Type one mean and SD values correspond to the first member of the combination, whereas type two values refer to the second one. The power for the analysis was defined as 0.8 and alpha was set to 0.05. Figure S3: Internal negative controls for cellular activation in response to anti‐IgM and anti‐IgG. The responses of the CD27^−^IgD^+^ (A) and CD27^+^IgD^+^ (B) populations to anti‐IgG, and responses of the CD27^−^IgD^−^ (C) and CD27^+^IgD^-^ (D) populations to anti‐IgM stimulation as an internal negative control. Figure S4: Dose–response characteristics of the circulating B cell subpopulations upon anti‐IgM and anti‐IgG stimulation. (A) The half‐maximal effect concentrations (EC50) of the anti‐IgM activating agent. (B) Statistical measures of the regression models on anti‐IgM stimulation. (C) The half‐maximal effect concentrations (EC50) of the anti‐IgG activating agent. (D) Statistical measures of the regression models on anti‐IgG stimulation. Nonlinear fitting, agonist vs response (three parameters), using the concentrations of the anti‐Ig antibodies and the maximum values of the kinetic measurements as response. Figure S5: Additional calcium kinetic parameters of the naïve and transitional, and unswitched B cell compartments A–F. Time and slope parameters extracted from the kinetic graphs of the naïve and transitional B cell compartments G–L. Time and slope parameters extracted from the kinetic graphs of the unswitched B cell compartment. Mann–Whitney test and Kruskal–Wallis nonparametric ANOVA with Dunn’s post hoc test. Figure S6: Additional calcium kinetic parameters of the naïve and transitional, and unswitched B cell compartments A–F. Time and slope parameters extracted from the kinetic graphs of the CD27^+^ switched memory B cell compartment B cell compartment G–L. Time and slope parameters extracted from the kinetic graphs of the and CD27^−^ switched memory B cell compartment. Mann–Whitney test and Kruskal–Wallis non‐parametric ANOVA with Dunn’s post hoc test.

## Data Availability

The authors confirm that the data supporting the findings of this study are available within the article and its Supporting Information. The code for the computational analysis can be found at the following GitHub link https://github.com/peterkaltenecker/CD38_in_B_cell_activation.
