# Supplementary material for: Linking CD38 Cell Surface Expression to the Unique Calcium Flux Properties in Human Peripheral B Lymphocytes Following anti‐IgM and anti‐IgG Stimulation
Source: J Immunol Res. 2026 Aug 27;2026:1377217. doi: 10.1155/jimr/1377217 (PMC13520786; doi:10.1155/jimr/1377217)
Supplement: Supplementary file 1 [file JIMR-2026-1377217-s001.zip › Supplementary Figure Legend.docx]

**S1 Fig. Gating pipeline.** **A.** Flow cytometric gating strategy to define the main circulating B cell compartments. **B.** FMO-based definition of CD38 expression in the main circulating B cell compartments. The full gating strategy, including the CD38-FMO boundaries, was applied per individual and per subpopulation, creating a compartment-specific threshold for CD38 positivity. The negative/positive gate boundaries were based on the resolution of negative populations and not set by chance or by a biased subjective approach. Contour plots were drawn in FlowJo using equal probability contouring using 5% threshold. At 5% probability there are no outliers outside the lowest contour line in the “CD27^-^ class-switched memory” population, thus, there is no error in picturing the events and the representation is consistent by using the same 5% threshold in all the plots. **C.** Double logistic representation of intracellular Ca^2+^ flux during B cell activation. **D.** Visual representation of marker stability by depicting the x/Time, y/fluorophore axes. The kinetic files were plotted as follows: x/Time, y/FSC-A. Two identical sections were selected at 200 sec and at 600 sec. The fluorescence intensity of the B cell markers was exported of these two gates. Median intensity of the B cell markers in a kinetic measurement from the 200 sec and 600 sec gates.

**S2 Fig. Power analysis.** Unpaired measurements comparing the main B cell compartments. Calculated effect sizes (d) and sample sizes (n) for the possible combinations of B cell subpopulations, in the case of maximum and AUC values, upon anti-IgM (**A**) and anti-IgG (**B**) activation. Type one mean and SD values correspond to the first member of the combination, whereas type two values refer to the second one. The power for the analysis was defined as 0.8 and alpha was set to 0.05.

**S3 Fig. Internal negative controls for cellular activation in response to anti-IgM and anti-IgG.** The responses of the CD27^-^IgD^+^ (**A**) and CD27^+^IgD^+^ (**B**) populations to anti-IgG, and responses of the CD27^-^IgD^-^ (**C**) and CD27^+^IgD^-^ (**D**) populations to anti-IgM stimulation as an internal negative control.

**S4 Fig. Dose-response characteristics of the circulating B cell subpopulations upon anti-IgM and anti-IgG stimulation. A.** The half maximal effect concentrations (EC50) of the anti-IgM activating agent. **B.** Statistical measures of the regression models on anti-IgM stimulation. **C.** The half maximal effect concentrations (EC50) of the anti-IgG activating agent. **D.** Statistical measures of the regression models on anti-IgG stimulation. Non-linear fitting, agonist vs. response (three parameters), using the concentrations of the anti-Ig antibodies and the maximum values of the kinetic measurements as response.

**S5 Fig. Additional calcium kinetic parameters of the naïve and transitional, and un-switched B cell compartments. A-F.** Time and slope parameters extracted from the kinetic graphs of the naïve and transitional B cell compartments. **G-L.** Time and slope parameters extracted from the kinetic graphs of the un-switched B cell compartment. Mann-Whitney test and Kruskal-Wallis non-parametric ANOVA with Dunn’s post hoc test.

**S6 Fig. Additional calcium kinetic parameters of the naïve and transitional, and un-switched B cell compartments.** **A-F.** Time and slope parameters extracted from the kinetic graphs of the CD27+ switched memory B cell compartment B cell compartment. **G-L.** Time and slope parameters extracted from the kinetic graphs of the and CD27- switched memory B cell compartment. Mann-Whitney test and Kruskal-Wallis non-parametric ANOVA with Dunn’s post hoc test.
